# Profiling of Polyphenol Composition and Antiradical Capacity of *Erica cinerea*

**DOI:** 10.3390/antiox6030072

**Published:** 2017-09-20

**Authors:** Alfredo Aires, Rosa Carvalho

**Affiliations:** 1 Centre for the Research and Technology for Agro-Environment and Biological Sciences, CITAB, Universidade de Trás-os-Montes e Alto Douro, UTAD, Quinta de Prados, 5000-801 Vila Real, Portugal; 2 Agronomy Department, Universidade de Trás-os-Montes e Alto Douro, UTAD, Quinta de Prados, 5000-801 Vila Real, Portugal; rpaula@utad.pt

**Keywords:** plants, phytochemicals, bioactive compounds

## Abstract

The aim of the current study was to determine the profile and content of polyphenols present in *Erica cinerea*, an important plant species from Northern Portuguese flora and often reported as having anti-inflammatory, antioxidant, and anti-radical activity. The analysis of polyphenols was performed by HPLC-DAD/UV-Vis, and the 2,2′-azinobis-(3-ethylbenzothiazoline-6-sulfonic acid) (ABTS•+) method was used to evaluate its radical scavenging activity. HPLC analysis showed that both plants presented a great diversity of compounds, with 33% flavones, 28% flavanols, and 26% hydroxycinnamic acids. The antiradical activity was dose-dependent, and the IC_50_ values were 0.251 mg mL^−1^. Based on our study, *E. cinerea* presented interesting bioactive compounds and it can be used to extract and purify bioactive polyphenols to be used in pharmaceutical or agro-food industries.

## 1. Introduction

*Erica cinerea* L. (Ericaceae) is largely present in the mountains of west and middle Europe, and is traditionally used in folk medicine to treat inflammatory diseases [1,2]. Several studies [3,4,5] have shown that Ericaceae contain a large number of bioactive compounds, including polyphenols. Additionally, recent studies with another medicinal Ericaceae species plant—*Erica australis* [6,7]—have shown that this plant is a rich source of polyphenols often associated with the prevention of degenerative and inflammatory diseases, as well as associated to antiradical processes [6,7]. In this context, we present this study in which we characterize the polyphenol composition of *E. cinerea* from Portuguese endemic flora by high performance liquid chromatography system coupled with a diode array type of UV/Vis detector (HPLC-DAD/UV-Vis) and determine its antiradical scavenging activity. The information will help to clarify if this plant species can be used to extract bioactive polyphenols.

## 2. Materials and Methods

### 2.1. Plant Material

One kilogram (fresh weight) of *Erica cinerea* (leaves and flowers) was collected in April 2015 in the natural open fields in Northern Portugal, Vila Real Region (400 m altitude) near the Natural Park of Alvão (N 41°17′35.538′′, W 7°44′29.6268′′). The samples were properly and botanically identified. After harvested and when in laboratory, the samples were dried in a freeze-drier system (Ultra Dry Systems TM, Warminster, PA, USA) and milled in a commercial blender and stored in dark flasks at 4 °C until extraction. The fresh and dry weights were recorded, and dry matter was determined. Three replications were taken.

### 2.2. Extraction

Ten milliliters of 70% aqueous methanol (methanol:water) was added to 100 mg of dry weight (dw) in screw cap tubes (10 mL) and mixed vigorously in a vortex (Genie 2, Fisher Scientific, Loughborough, Leicestershire, UK), heated at 70 °C (1083, GFL-Gesells chaft ffur Labortechnik GmbH, Burgwedel, Germany) during 20 min, and agitated every 5 min. The extracts were then centrifuged at 4000 rpm for 15 min (Kubota, Tokyo, Japan). The supernatants were filtered, firstly through a Whatman No. 1 paper and then through Polytetrafluoroethylene (PTFE) 0.2 µm, Ø 13 mm (Teknokroma, Sant Cugat del Vallés, Barcelona, Spain) filters to amber HPLC vials (Chromabond 2-SVW(A) ST-CPK, Sigma-Aldrich, Tauferkichen, Germany). The final extracts were stored under refrigeration (−20 °C) until HPLC analysis and antiradical colorimetric determination.

### 2.3. Polyphenol Composition by HPLC-DAD/UV-Vis

The quantification of polyphenols present in *E. cinerea* extracts was performed using a HPLC-DAD-UV/Vis [8] system equipped with a C18 column (250 × 46 mm, 5 µm) (ACE^®^ HPLC columns, Advanced Chromatography Technologies, Ltd., Aberdeen, Scotland), an eluent composed by 1% of trifluoroacetic acid (TFA) in water (solvent A) and 1% TFA in acetonitrile (solvent B). The elution was performed with a flow rate of 1 mL min^−1^, a gradient starting with 100% water, and an injection volume of 10 μL. The chromatograms were recorded at 254, 280, 320, 370, and 520 nm. The polyphenols were identified and quantified using peak retention time, UV spectra, and UV max absorbance bands and trough comparison with external commercial standards (Extrasynthese, CEDEX, France, and Sigma-Aldrich, Tauferkichen, Germany), as well as by comparing with published literature [9,10]. The external standards were freshly prepared in 70% methanol (70 methanol:30 water) at a concentration of 1.0 mg mL^−1^ and running in HPLC-DAD-UV-Vis before the samples. The results were expressed as µg g^−1^ dry weight (dw). All solvents were HPLC-grade solvents.

### 2.4. ABTS (2,2′-Azinobis-(3-Ethylbenzothiazoline-6-Sulfonic Acid)) Radical Scavenging Activity

The ABTS radical scavenging activity was evaluated using the (ABTS•+) radical-scavenging activity colorimetric method [11] conducted in a 96-well microplate. Ten different methanolic concentrations (ranging from 0.195 to 10.0 mg mL^−1^) of *E. cinerea* extracts were prepared and used in the antiradical assay. A radical ABTS solution was freshly prepared by mixing 7 mM of ABTS at pH 7.4 (5 mM NaH_2_PO_4_, 5 mM Na_2_HPO_4_, and 154 mM NaCl) with 2.5 mM potassium persulfate, followed by storage in the dark at room temperature for 16 h. The mixture was then diluted with ethanol to give an absorbance of 0.70 ± 0.02 units at 734 nm, using a multiscan microplate reader (Multiskan™ FC Microplate Photometer, Waltham, MA, USA). After that, to each microplate well 15 µL of extract was added followed by addition of 285 µL fresh ABTS solution. The microplates were then incubated at room temperature in the dark for 10 min. The absorbance values were then measured in a multiscan microplate reader at 734 nm. Simultaneously, a curve with commercial standard ascorbic acid was used and served as positive control of antiradical activity. The results were expressed as percentage (%) of ABTS radical scavenging activity, using the following formula: ABTS radical scavenging (%) = [(absorbance solvent − absorbance sample)/absorbance solvent) × 100]. The concentration of antioxidants which scavenge the free radical ABTS•+ about 50% (IC_50_) for plant extract and ascorbic acid was also determined. In order to classify the antiradical activity, we selected the following scale: IC_50_ < 0.1 mg mL^−1^—strong/high antiradical activity; 0.1 mg mL^−1^ < IC_50_ < 1 mg mL^−1^—moderate; and >1 mg mL^−1^—weak antiradical activity.

### 2.5. Statistical Analysis

All determinations were carried out in triplicate, and the results were expressed as mean values ± standard deviation (SD). The Software SPSS v.17 (SPSS-IBM, Orchard Road-Armonk, New York, NY, USA) was used to carry out these analysis.

## 3. Results

The analysis by *HPLC-DAD/UV-Vis* of *E. cinerea* methanolic extracts revealed the presence of different classes of polyphenols (Table 1). The quantity of each polyphenol identified in each plant extract is presented in Table 1. *E. cinerea* exhibited an average level of 1646 µg g^−1^ dw, in which the highest values were found for chlorogenic acid + neochlorogenic acid (22%), luteolin-3-*O*-rutinoside (11%), luteolin-3-*O*-galactoside (10%), apigenin-7-*O*-rutinoside (9%), and quercetin-3-*O*-glactoside (6%). These polyphenols represented more than 50% of the total polyphenols identified.

**Figure 1:**
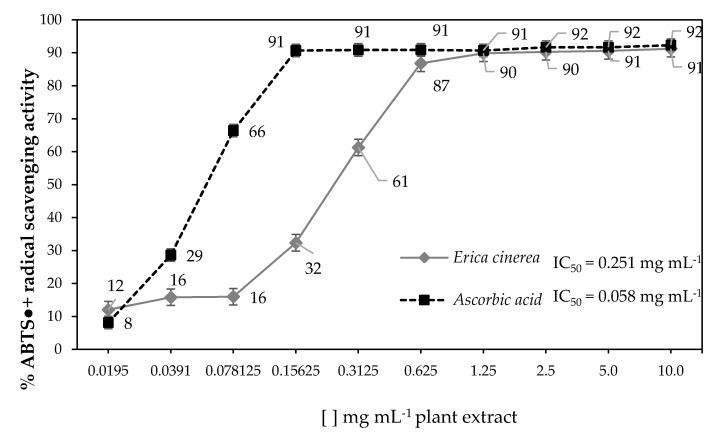
ABTS (2,2′-azinobis-(3-ethylbenzothiazoline-6-sulfonic acid)) radical scavenging activity of *E. cinerea* and ascorbic acid at different concentrations, and respective IC_50_ values.

Figure 1 shows the variation of antiradical activity by ABTS method, including the respective IC_50_ values (samples with lower IC_50_ values correspond to those with stronger antiradical activity), and it was possible to observe that the extracts of *E. cinerea* presented lower antiradical activity when compared to ascorbic acid. However, when compared to other medicinal and aromatic plants [12,13,14], and with the scale adopted, we considered the antiradical activity moderate. 

## 4. Discussion

Ericaceae plants have been presented as having health protective effects due to their richness in polyphenols—particularly phenolic acids and flavonoids, highly associated with high antioxidant capacity, antiradical activity, and anti-inflammatory effect [6,7]. Among these compounds, polyphenols like chlorogenic acid, caffeic acid, kaempferol, myricetin, quercetin, and luteolin glycosides [15,16,17,18,19] have been reported as having important health potential, since they present high capacity to scavenge different oxidizing molecules (such as superoxide anion, hydroxyl radical, or peroxy radicals, and as quenchers of singlet oxygen) or to increase powerful intracellular antioxidant enzymes and act as pro-active cell protectors [15,16,17,18,19]. Thus, a high content of such polyphenols should mean high bioactivities. Therefore, based on our results, since *E. cinerea* contains high levels of such important bioactive polyphenols, we may state that *E. cinerea* shows high antioxidant potential, which may partially explain the ethnobotanical use of this plant as a treatment for inflammation-related conditions. 

## 5. Conclusions

The richness of *Erica cinerea* in polyphenols like hydroxycinnamic acids, flavanols, flavonols, and flavones justify their use in traditional and folk medicine, and they can be used to extract and purify important bioactive compounds for the nutraceutical and food supplement market. More studies measuring seasonal or geographical variations in the polyphenols profile and content of *E. cinerea* and exploring if this plant can be a good medical crop plant should be done in the future.

## Figures and Tables

**Table 1:** Polyphenols and respective retention time (Rt), maximum absorption (λmax), and quantities in methanolic extracts of *Erica cinerea* (by elution order). ^1^

Polyphenols	Rt (min)	UV Detection (nm)	UV λmax (nm)	Quantity (µg g^−1^ dw)	Percentage in the Total (%)
*trans*-Cinnamic acid	15.05	320	274,318	5.0 ± 0.1	0.3
Neochlorogenic acid	15.94	320	308,322	104.4 ± 0.2	6.3
Epigallocatechin	16.55	280	279	21.7 ± 0.9	1.3
5-*O*-Caffeoylquinic acid	17.58	320	284,311	10.0 ± 0.9	0.6
Chlorogenic acid	17.86	320	303,322	240.7 ± 1.1	14.6
Catechin	18.00	320	279	18.5 ± 0.1	1.1
Epicatechin	18.36	280	280	29.8 ± 0.4	1.8
Epigallocatechin gallate	18.55	280	279	18.8 ± 3.3	1.1
Gallocatechin gallate	18.75	280	279	8.0 ± 0.6	0.5
Myricetin-3-*O*-rutinoside	19.04	370	275,339,380	28.6 ± 1.0	1.7
Cyanidin-3-*O*-rutinoside	19.18	520	237,279,517	14.0 ± 0.2	0.9
Caffeic acid	19.31	320	280,324	34.3 ± 1.5	2.1
Epicatechin gallate	19.51	280	279	36.7 ± 0.0	2.2
Cyanidin-3-*O*-arabinoside	20.39	520	237,279,518	1.9 ± 0.1	0.1
Kaempferol-3-*O*-galactoside	20.59	370	266,355	25.6 ± 0.9	1.6
Myricetin-3-*O*-glucoside	20.74	370	271,354	7.7 ± 0.4	0.5
Myricetin-3-*O*-galactoside	21.17	370	273,358	9.9 ± 0.3	0.6
Quercetin-3-*O*-galactoside	21.34	370	254,358	104.2 ± 0.2	6.3
Gossypetin	21.63	370	273,356	19.8 ± 0.3	1.2
Hesperidin	21.85	280	285,336	26.0 ± 0.3	1.6
Luteolin-3-*O*-rutinoside	22.02	370	253,350	182.0 ± 0.1	11.1
Luteolin-3-*O*-glucoside	22.15	370	254,351	57.7 ± 1.6	3.5
Quercetin-3-*O*-glucoside	22.60	370	256,359	92.2 ± 3.0	5.6
Kaempferol-3-*O*-glucoside	23.03	370	254,355	73.4 ± 0.9	4.5
Petunidin	23.19	520	278,530	37.4 ± 0.1	2.3
Luteolin-3-*O*-galactoside	23.31	370	269,346	163.7 ± 0.3	9.9
Rosmarinic acid	23.55	320	282,328	26.2 ± 0.4	1.6
Apigenin-7-*O*-rutinoside	24.13	370	265,333	145.7 ± 1.1	8.9
Isorhamnetin-3-*O*-rutinoside	24.92	370	265,342	57.1 ± 3.2	3.5
Quercetin-3-*O*-rhamnoside	28.02	370	255,369	45.2 ± 3.7	2.7

^1^ Values expressed as mean ± standard deviation (SD) of three replicates.
